# Paced breathing causes tonic change rather than phasic modulation of superficial venous diameter

**DOI:** 10.1186/s40101-025-00392-9

**Published:** 2025-05-16

**Authors:** Miharu Matsumoto, Nobuko Hashiguchi, Hiromitsu Kobayashi

**Affiliations:** 1 Department of Health Sciences, Kyushu University, 3 - 1- 1, Maidashi, Higashi-Ku, Fukuoka, 812 - 8582 Japan; 2 Ishikawa Prefectural Nursing University, 1-1, Gakuendai, Kahoku, Ishikawa, 929 - 1210 Japan

**Keywords:** Peripheral superficial vein, Venipuncture, Respiratory modulation, Ultrasound imaging

## Abstract

**Background:**

Respiratory modulation is generally observed in the inferior vena cava (IVC). If similar respiratory modulation exists in peripheral superficial veins, it would be possible to dilate the vein diameter by respiratory control. This may improve the success rate of venipuncture in clinical practices. Hence, the present study aimed to investigate the respiratory modulation in peripheral superficial veins.

**Methods:**

This study included 21 healthy female volunteers (mean age 21.8 ± 0.9 years). Participants performed spontaneous breathing (SB) and paced breathing (PB). B-mode ultrasound imaging was used to continuously monitor the cutaneous veins of the left elbow fossa for 50 s.

**Results:**

Vein diameter demonstrated a clear modulation consistent with paced breathing, and the amplitude of vein diameter modulation was greater at 10 s-PB than at 3 s-PB. Additionally, PB affected the baseline of modulation (mean vein diameter). The baseline exhibited the largest diameter in SB, followed by 3 s-PB and 10 s-PB. The baseline for SB and 10 s-PB demonstrated a statistically significant difference (*p* = 0.03). Respiratory modulation was confirmed in peripheral superficial veins; however, tonic change in baseline diameter was dominant over phasic modulation. Even when vein diameter was most dilated at 10 s-PB, the diameter at that time was smaller than the mean diameter at SB.

**Conclusions:**

This study demonstrated that the peripheral superficial vein diameter exhibited respiratory modulation, similar to the IVC. Although respiratory modulation of the IVC has been well documented in previous studies, the present findings provide novel evidence of this phenomenon in the peripheral superficial veins. Additionally, this study identified tonic changes in the mean vein diameter, which were more dominant than phasic modulations. Furthermore, the mean vein diameter during SB was greater than the maximum diameter observed during 10 s-PB. These findings suggested that PB for 50 s during venipuncture did not enhance venous access.

## Background

Venipuncture is an invasive procedure performed for blood collection or venous catheter insertion. Poor palpability and visibility are prevalent characteristics of veins diagnosed with failed venipuncture [1–4]. Both visibility and palpability are associated with vein diameter [5]. In particular, vein diameter constitutes a crucial factor in venipuncture success rate. This is consistent with previous results that larger vein diameters are associated with higher venipuncture success rates [6–8].

Local heating has been used in clinical practice to dilate veins and improve success rates. Local heating improves venous palpability and visibility [9–12], thereby significantly increasing the success rate of initial venipuncture attempts [9, 12]. Condition-controlled experimental studies have revealed that local heating dilated the vein diameter by 2–22% [13–15].

Local heating duration depends on the situation. In particular, 70% of clinicians reported it as approximately 2–5 min in clinical practice [16], whereas experimental studies have used 7–15 min of heating [13–15]. Therefore, vein dilation by local heating requires at least several minutes.

Respiration generally influences the diameter and blood flow dynamics of the inferior vena cava (IVC), internal jugular vein, upper extremity veins, such as the subclavian vein (SCV), and axillary veins [17–21]. Respiratory modulation induces vein contraction during inspiration and dilatation during expiration [22, 23]. The respiratory modulation magnitude depends on the depth of breathing. Deeper breathing generates larger vein modulation [24, 25]. Furthermore, central veins (e.g., IVC) have a large respiratory modulation, and the modulation degree tends to decrease from the center to the periphery [26]. However, previous studies have not included veins that are more peripheral than the axillary vein, and respiratory modulation in the superficial veins of the forearm remains unknown. Vein diameters could be dilated without heating for a few minutes if the respiratory modulation is observed in peripheral superficial veins as well as IVC. This approach is beneficial, particularly for patients with difficult venous access. Therefore, the present study investigated the respiratory modulation in peripheral superficial veins.

## Methods

### Participants

This study included 21 young and healthy female participants (mean age 21.8 ± 0.9 years). The exclusion criteria were patients with a disease under treatment, pregnant or possibly pregnant, and smokers. The specific number of participants was identified based on a previous study using a similar methodology for imaging analysis [25]. Female generally have smaller vein diameters than men [27] and venipuncture is generally more difficult; thus, this study only included female. The mean height, weight, and body mass index were 158.1 ± 4.9 cm, 51.0 ± 6.9 kg, and 20.4 ± 2.5 kg/m^2^, respectively. The participants had an average BMI similar to that of females in Japan aged 20–29 years published by the Japanese Ministry of Health, Labour and Welfare [28]. Participants were instructed to abstain from alcohol and sleep for at least 6 h and were restricted from strenuous exercise the day before the experiment.

### Procedures

This study was conducted in August 2023. Participants were seated and rested for at least 30 min after entering the controlled environment chamber. Room conditions were maintained at 25 °C with a relative humidity of 50%. Participants were then trained to control their breathing by synchronizing with an audio signal presented by a computer program. This computer program demonstrates auditory signals for the onset of inspiration or expiration. Breathing was controlled in three patterns: spontaneous breathing (SB), 3 s paced breathing (3 s-PB), and 10 s paced breathing (10 s-PB). The 3 s-PB corresponds to the respiratory rate during spontaneous breathing, whereas the 10 s-PB equates to the resonance frequency of heart rate variability (HRV) at which respiratory sinus arrhythmia (RSA) demonstrates maximal amplitude [29–31]. These three conditions were randomized, and each breath was consecutively performed for 50 s.

The forearm was positioned on a platform at the heart level. The elbow joint was aligned with the mark on the table, the forearm was kept extended, and the arm was fastened with a belt to avoid movement. The nondominant elbow fossa vein, which is easiest to delineate with ultrasound equipment, was selected. The height and angle from the probe to the skin were replicated each time by attaching the probe to the table.

**Fig. 1:**
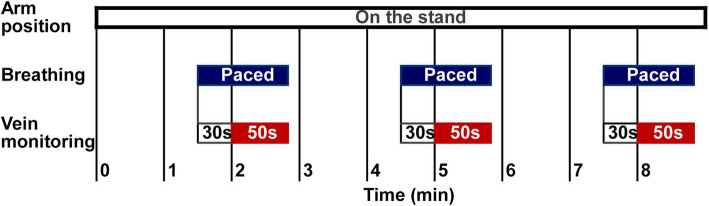
Experimental protocol. Monitoring of peripheral vein diameters started after 2 min with the arm on the stand and continued for 50 s. The arm was taken off after three repeated measurements, and a 2-min break was permitted. The same procedure was repeated thrice

Figure 1 shows the experimental protocol. A quiet environment was maintained in all measurements. Measurements were repeated thrice consecutively per breathing condition (SB, 3 s-PB, and 10 s-PB), during which the upper arm was placed on a table and remained fixed. The forearm was removed from the table and rested for 2 min after completing the three consecutive measurements. Subsequently, the same procedure was repeated, and measurements were obtained under all breathing conditions.

### Measurements

An ultrasound imaging device (Versana Active; GE Health Care, Tokyo, Japan) was utilized to obtain images of the median cutaneous vein of the left elbow fossa. The B-mode short-axis view was selected due to the mode’s reduced sensitivity to slight arm movements in comparison to the M-mode view. All images were acquired by the same operator with the same settings (frequency 12 MHz, image depth 20 mm, dynamic range 51 dB, and time gain 56 dB). To prevent compression of the vein by the probe, a gel layer approximately 1 cm thick was placed on the subject's forearm. The images were recorded continuously as a movie file.

The breathing of the participants was continuously monitored using a thermocouple sensor attached to the nostrils during the experiment. The output of the thermocouple sensor was converted to a digital signal at a sampling frequency of 100 Hz (Thermoception Analyzer Intercross- 210, Intercross Inc., Tokyo, Japan).

### Image analysis

**Fig. 2:**
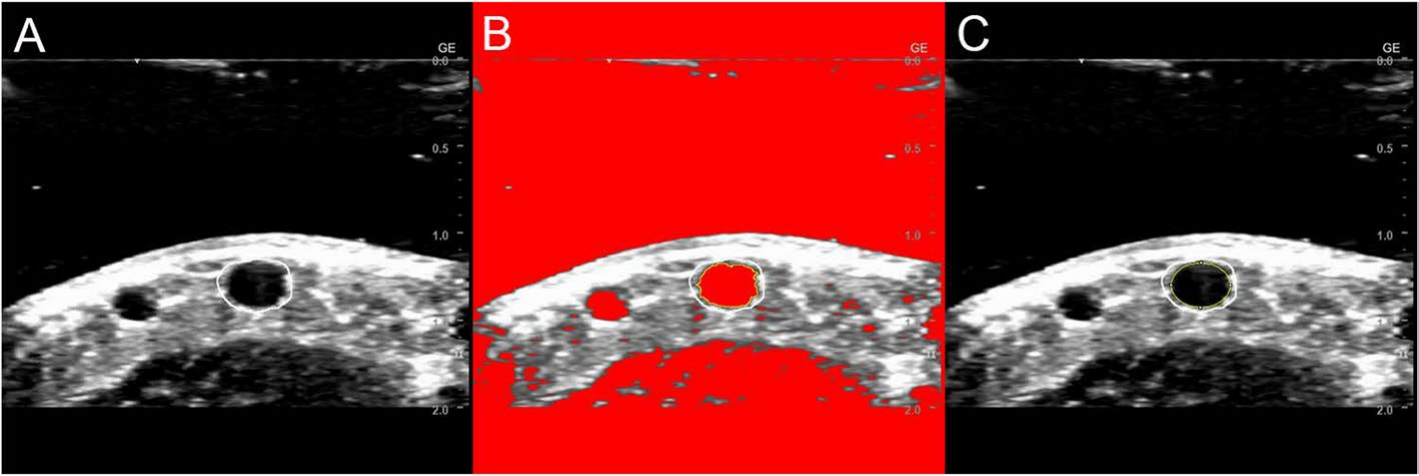
Image analysis. The image was converted to 8-bit grayscale. The region of interest was positioned using a manual tool (**A**). Edge detection (**B**) and ellipse fitting (**C**) were automatically performed with ImageJ

Figure 2 shows the analysis. The captured images were divided into tiff files at 1 s intervals and imported into the image analysis software, ImageJ [32]. The captured images were first converted to an 8-bit grayscale, and then the region of interest was manually positioned. Additionally, the binarization process was used to detect edges. Most images were processed with a threshold of 70. However, a few instances of edges that could not be extracted underwent manual optimization on an image-by-image basis. The detected edges were fitted with ellipses, and the short diameter was automatically calculated from the ellipse’s area (mm^2^).

### Statistical analysis

The vein diameter and respiratory rate data recorded every second were ensemble-averaged. The ensemble average reduces noise other than paced breathing, thereby facilitating capturing data features [33, 34]. This technique is frequently used for time series analyses of physiological data [35–40]. In this case, the 3 s- and 10 s-PB were analyzed by aligning the start of the inspiration. To examine the trend over time, correlation analyses were performed on the ensemble average of the vein diameter. The respiratory rate during SB was determined by counting the number of peaks in the waveform of the nostril temperature.

Frequency analysis was conducted to quantify the magnitude of respiratory modulation in the vein diameter. The power spectrum was collected by analyzing the vein diameter variation for 50 s using the maximal entropy method with the seventh-order autoregressive model. The natural logarithm of the integrated power of 0.099–0.101 Hz for 10 s-PB s and 0.332–0.334 Hz for 3 s-PB represented the magnitude of the variability for each condition. Three repetitions of the same condition were averaged while testing for differences between breathing conditions, and a paired *t*-test was performed on the data collected from 21 participants.

The average vein diameters over 50 s were determined and defined as the baseline of the modulation (baseline). Repeated-measure one-way analysis of variance was performed on data gathered from the 21 participants to identify differences between respiratory conditions. Additionally, the Bonferroni method was utilized for multiple comparisons, and differences with a *p*-value of < 0.05 were considered statistically significant. R version 4.2.2 for Windows was used for statistical analysis.

### Ethics approval and consent to participate

This study followed the ethical principles stated in the Declaration of Helsinki and obtained ethical approval from the Clinical Research Ethics Committee of Kyushu University Hospital (Approval no. 23081–00). All study participants signed written informed consent forms.

## Results

**Fig. 3:**
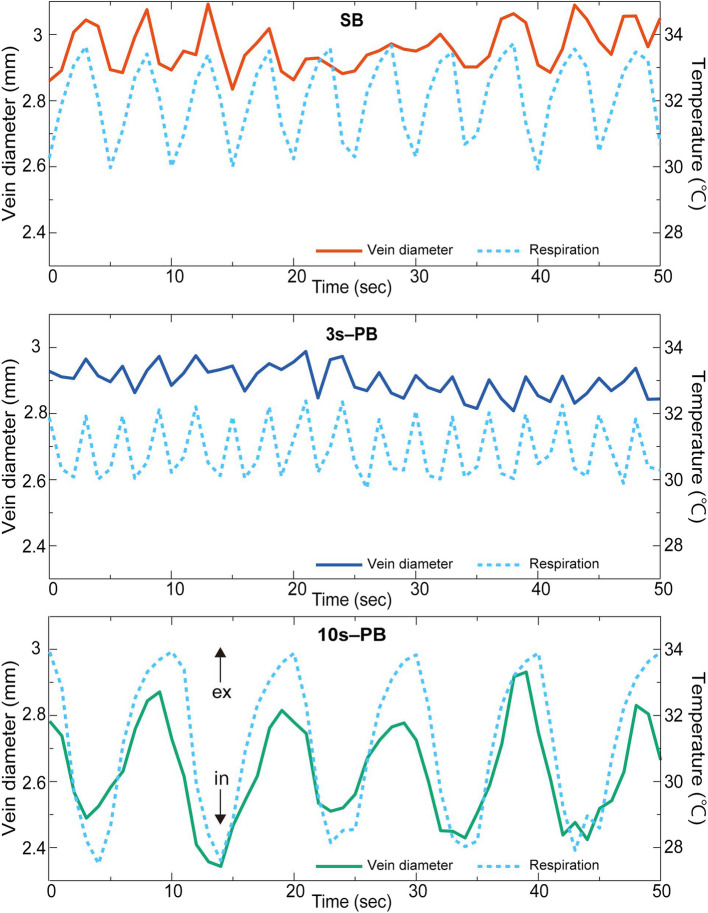
Examples from participant #17 with vein diameter and respiration. Dynamic changes in respiration and vein diameter of one participant. Vein diameter modulation was consistent with respiration. The horizontal axis represents time and the vertical axis denotes vein diameter (left) and the temperature change around the perinasal (right). The solid line indicates the vein diameter and the dashed line represents respiration. Perinasal temperature decreases with inspiration and increases with expiration. The upper panel shows SB and the middle and lower panels demonstrate 3 s-PB and 10 s-PB, respectively

Figure 3 illustrates examples of dynamic changes of vein diameter and respiration in one participant. The participant breathed at approximately a 5-s cycle (0.2 Hz) in the SB condition and breathed at the designated cycles (3 and 10 s) in the PB conditions. The vein diameter demonstrated modulation consistent with the respiratory cycle under the three respiratory conditions. Respiratory modulation in vein diameter was particularly clear and exhibited large amplitude at 10 s-PB compared with SB and 3 s-PB.

**Fig. 4:**
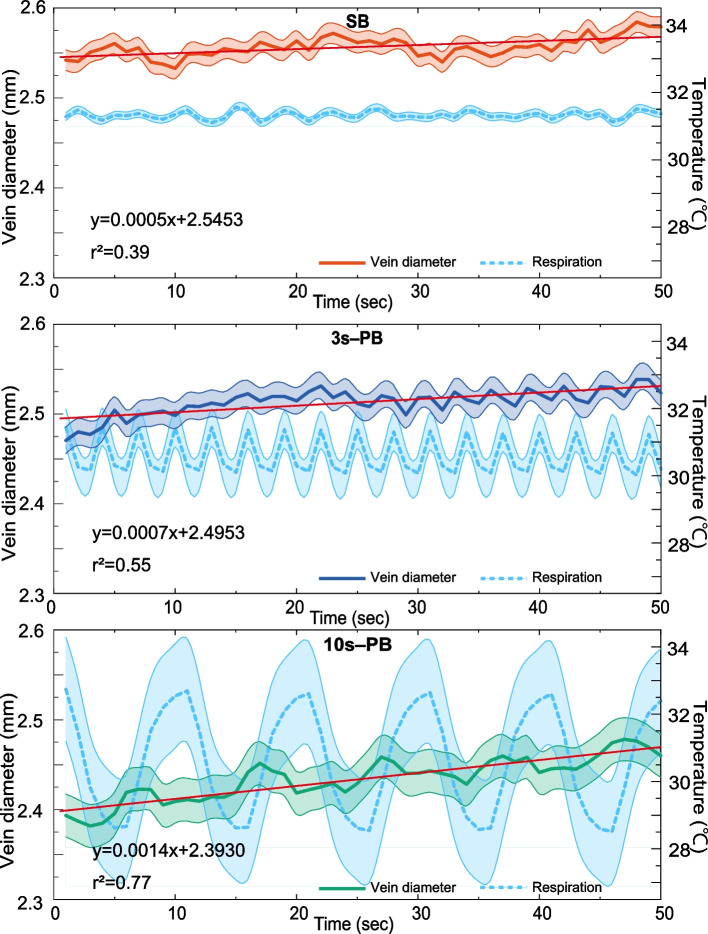
Ensemble average of the respiratory modulation of vein diameter. Fifty-second recordings of vein diameter averaged over 21 participants. The three panels show the results for SB, 3 s-PB, and 10 s-PB, respectively. The upper trace (solid line) in each panel indicates the vein diameter, and the lower trace (dashed line) shows the respiration. Error bars represent standard deviations (SDs)

Figure 4 illustrates the ensemble average of vein diameter for the 21 participants. The participants breathed in 2–8 s cycles (average of approximately 3.9 s) for SB. The ensemble average of SB demonstrated a nearly flat response because the breathing phases of each participant were not in phase. In contrast, the 3 s- and 10 s-PBs exhibited vein diameter modulation synchronized with respiration even in the ensemble average, and the modulation amplitude was greater during 10 s-PB than during 3 s-PB. These three breathing conditions expressed their influence as a tonic change in baseline diameter rather than a phasic modulation. The baseline venous diameter was largest in the SB and smallest in the 10 s-PB.

Linear correlation analysis demonstrated an association, with coefficients of determination (r2) of 0.39, 0.55, and 0.77 for SB, 3 s-PB, and 10 s-PB, respectively.

**Fig. 5:**
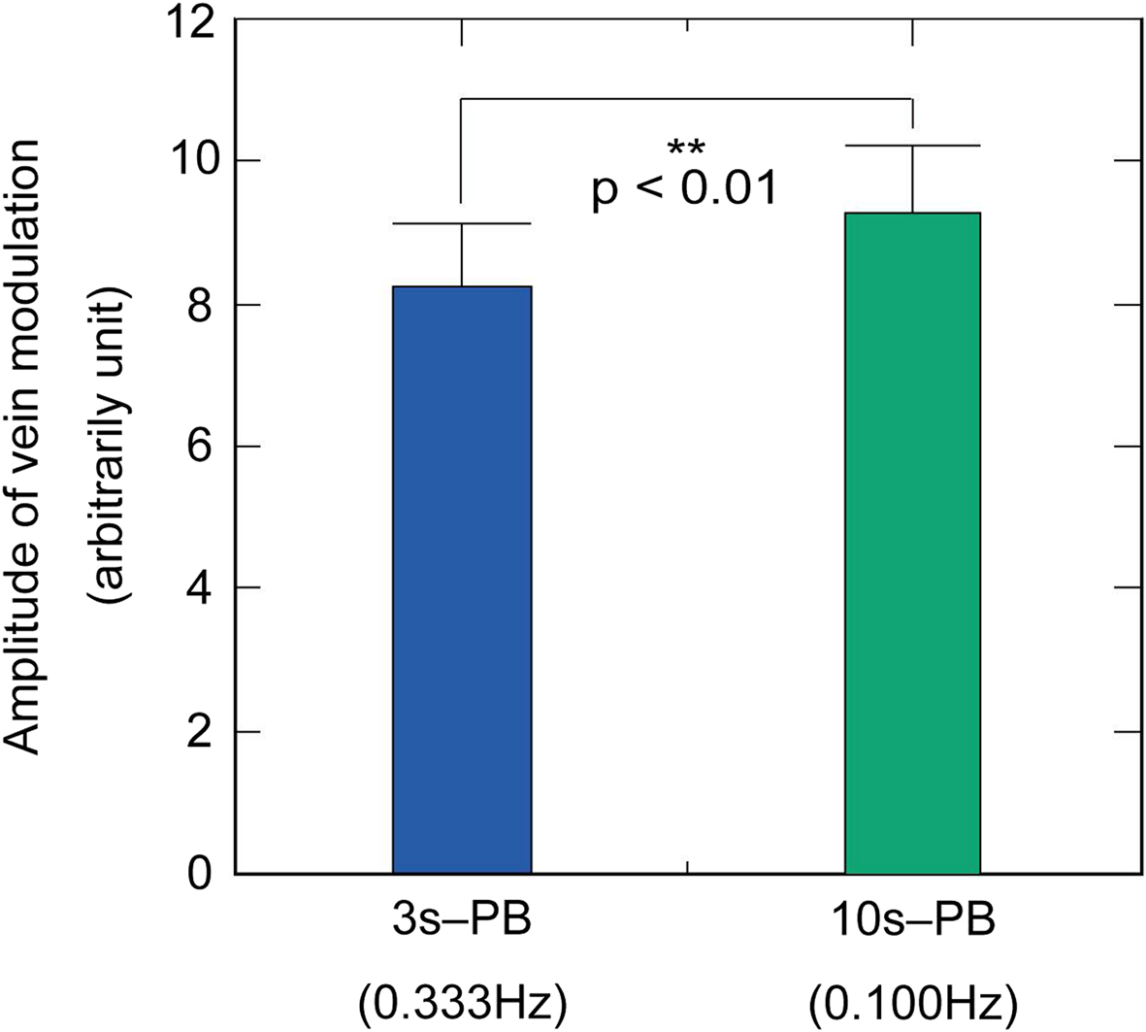
Effect of breathing period on the spectral power of vein modulation (*n* = 21). Comparison of the average spectral power of vein modulation of 3 s-PB and 10 s-PB. The vein modulation induced by 10 s-PB was significantly larger than that by 3 s-PB. Error bars represent SDs

Figure 5 illustrates a comparison of the magnitude of vein diameter modulation that coincides with the breathing cycle. The vein modulation induced by 10 s-PB was significantly larger (*p* < 0.01) than that by 3 s-PB.

**Fig. 6:**
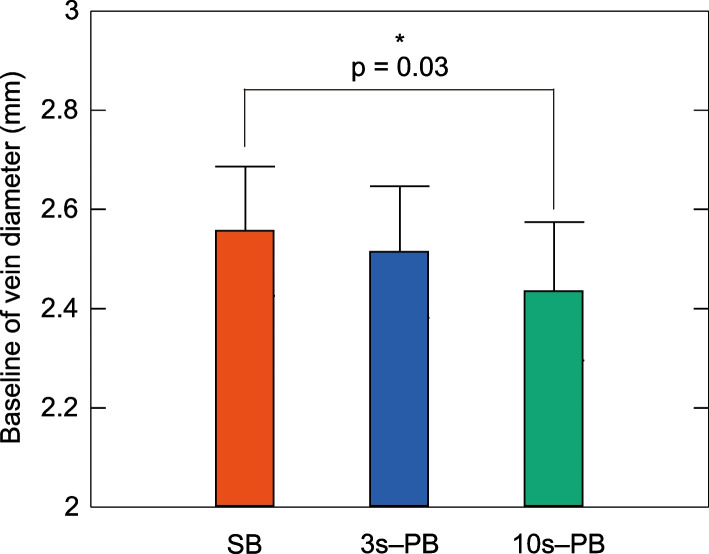
Baseline comparison of vein diameters (*n* = 21). Baseline comparison of vein diameter at SB (orange), 3 s-PB (blue), and 10 s-PB (green). Error bars denote standard error

Figure 6 shows a comparison of baselines (averaged over 50 s) of vein diameters. The mean baseline vein diameter values were 2.55 ± 0.01 mm, 2.51 ± 0.01 mm, and 2.43 ± 0.02 mm for SB, 3 s-PB, and 10 s-PB, respectively. The average value was smaller for PBs compared to SB. The one-way analysis of variance revealed a statistically significant difference between the three groups, and the multiple comparison tests demonstrated a significant difference between SB and 10 s-PB (p = 0.03).

## Discussion

### Respiratory modulation in peripheral superficial veins

The ensemble average of vein diameter demonstrated periodic fluctuations synchronized with respiration under PB conditions of this study. Vein diameters demonstrated similar responses to respiration with RSA in HRV. The RSA is generally a frequency-dependent response, indicating a maximum amplitude at approximately 0.1 Hz [29–31]. The amplitude of the periodic fluctuation in the vein diameter was greater in 10 s-PB than in 3 s-PB, similar to RSA (*p* < 0.01). The present results indicated that the amplitude of the respiratory modulation of the peripheral vein diameter depends on the respiratory frequency. Hemodynamic changes due to intrathoracic or intra-abdominal pressure fluctuations represent a possible mechanism for the modulation synchronized with breathing. In other words, inspiration triggers veinous contraction due to increased venous return, which is facilitated by decreased intrathoracic pressure; conversely, expiration causes vein diameter dilation [17–21]. This phenomenon has been widely observed in the IVC, IJV, SCV, and axillary veins [17–21]. Furthermore, autonomic nervous system activity [41, 42] and vascular smooth muscle contraction [43] may be involved in venoconstriction. It is likely that these factors are complexly interconnected.

A decreased breathing pace is generally expected to increase tidal volume. The deeper respiration has been known to produce a greater RSA amplitude [30]. A similar response was reported in the respiratory modulation of the central vena cava such as IVC and SCV [21, 24, 25]. However, RSA enhancements by low frequency breathing are not solely due to an increase in tidal volume, and larger amplitude in RSA occurs at lower frequency despite constant tidal volume [29, 30]. Therefore, the increase in the amplitude of respiratory modulation in the vein is also considered independent of the tidal volume.

### Tonic change in the vein diameter

As expected, in this study, PB induced a phasic modulation of the vein diameter. On the other hand, PB also induced tonic changes in the vein diameter; compared with SB, 10 s-PB reduced the mean vein diameter by 5%. A trend toward smaller mean vein diameters was also noted in 10 s-PB than that in 3 s-PB; however, this difference was not significant.

In addition, vein diameters during PB exhibited a linear increasing trend over time that was not synchronized with respiration (Fig. 4). These tonic changes in the vein diameter were unexpected because, in HRV, PB significantly alters the phasic amplitude, whereas the tonic component (e.g., mean heart rate) is unaffected by PB [44, 45]. If the increasing trend persists for several minutes, 10 s-PB may dilate the periferal veins. This increasing trend is a remarkable phenomenon.

An previous study (Eckstein et al., 1958) reported that hyperventilation causes venoconstriction of the forearm [46]. Therefore, PB may affect the vein diameter. Changes in blood pH levels may cause the tonic changes observed in this study. However, as the blood pH level was not measured in this study, determining the direct effect of pH changes on the vein diameter was not possible. Measuring changes in the blood pH level during PB and investigating its effect on vein diameter are warranted. The results of this study suggested the significance of considering the effects of respiration in measuring vein diameter and offer novel insights for future research.

Moreover, the results of this study raised new questions. Under SB condition, participants breathed spontaneously with an average cycle of approximately 3.9 s. If tonic changes in vein diameter were merely influenced by breathing frequency, the mean vein diameter in SB should lie between 3 s-PB and 10 s-PB. However, the largest vein diameters were obtained in SB condition. The results of the present study suggested that the tonic changes in vein diameter with PB may not be solely due to respiratory frequency. Further studies are needed to clarify the mechanism of PB-induced tonic changes in vein diameter.

## Conclusion

This study revealed that the peripheral superficial vein diameter exhibited respiratory modulation, similar to the IVC. Vein diameters contracted with inspiration and dilated with expiration. Furthermore, the amplitude of the vein diameter modulation was observed to be respiratory frequency-dependent. Previous studies have documented respiratory modulation in the IVC; however, the respiratory modulation in the peripheral superficial veins represents a novel finding of this study.

The tonic modulation of the mean vein diameter constitutes another finding of this study. Tonic changes in the mean vein diameter were dominant rather than phasic modulation. The mean vein diameter during SB was larger than the largest vein diameter during 10 s-PB. Therefore, PBfor 50 s during venipuncture cannot improve venous access.

## Data Availability

The datasets during and/or analyzed during the current study available from the corresponding author on reasonable request.

## Abbreviations

HRV: Heart rate variability
IJV: Internal jugular vein
IVC: Inferior vena cava
PB: Paced breathing
RSA: Respiratory sinus arrhythmia
SB: Spontaneous breathing
SCV: Subclavian vein
